# Prevalence of *Salmonella* in Stool During the Vaccine Impact on Diarrhea in Africa (VIDA) Study, 2015–2018

**DOI:** 10.1093/cid/ciac985

**Published:** 2023-04-19

**Authors:** Irene N Kasumba, Helen Powell, Richard Omore, M Jahangir Hossain, Samba O Sow, John Benjamin Ochieng, Henry Badji, Jennifer R Verani, Marc-Alain Widdowson, Sunil Sen, Shamima Nasrin, Jasnehta Permala-Booth, Jennifer A Jones, Anna Roose, Dilruba Nasrin, Ciara E Sugerman, Jane Juma, Alex Awuor, Joquina Chiquita M Jones, Sanogo Doh, Catherine Okoi, Syed M A Zaman, Martin Antonio, Elizabeth Hunsperger, Clayton Onyango, James Platts-Mills, Jie Liu, Eric Houpt, Kathleen M Neuzil, Karen L Kotloff, Sharon M Tennant

**Affiliations:** Center for Vaccine Development and Global Health, University of Maryland School of Medicine, Baltimore, Maryland, USA; Department of Medicine, University of Maryland School of Medicine, Baltimore, Maryland, USA; Center for Vaccine Development and Global Health, University of Maryland School of Medicine, Baltimore, Maryland, USA; Department of Pediatrics, University of Maryland School of Medicine, Baltimore, Maryland, USA; Center for Global Health Research, Kenya Medical Research Institute, Kisumu, Kenya; Medical Research Council Unit, The Gambia at the London School of Hygiene & Tropical Medicine, Banjul, The Gambia; Centre pour le Developpement des Vaccins (CVD-Mali), Bamako, Mali; Center for Global Health Research, Kenya Medical Research Institute, Kisumu, Kenya; Medical Research Council Unit, The Gambia at the London School of Hygiene & Tropical Medicine, Banjul, The Gambia; Division of Global Health Protection, US Centers for Disease Control and Prevention, Nairobi, Kenya; Division of Global Health Protection, US Centers for Disease Control and Prevention, Nairobi, Kenya; Center for Vaccine Development and Global Health, University of Maryland School of Medicine, Baltimore, Maryland, USA; Department of Medicine, University of Maryland School of Medicine, Baltimore, Maryland, USA; Center for Vaccine Development and Global Health, University of Maryland School of Medicine, Baltimore, Maryland, USA; Department of Medicine, University of Maryland School of Medicine, Baltimore, Maryland, USA; Center for Vaccine Development and Global Health, University of Maryland School of Medicine, Baltimore, Maryland, USA; Department of Medicine, University of Maryland School of Medicine, Baltimore, Maryland, USA; Center for Vaccine Development and Global Health, University of Maryland School of Medicine, Baltimore, Maryland, USA; Department of Medicine, University of Maryland School of Medicine, Baltimore, Maryland, USA; Center for Vaccine Development and Global Health, University of Maryland School of Medicine, Baltimore, Maryland, USA; Department of Pediatrics, University of Maryland School of Medicine, Baltimore, Maryland, USA; Center for Vaccine Development and Global Health, University of Maryland School of Medicine, Baltimore, Maryland, USA; Department of Medicine, University of Maryland School of Medicine, Baltimore, Maryland, USA; Division of Foodborne, Waterborne, and Environmental Diseases, US Centers for Disease Control and Prevention, Atlanta, Georgia, USA; Center for Global Health Research, Kenya Medical Research Institute, Kisumu, Kenya; Center for Global Health Research, Kenya Medical Research Institute, Kisumu, Kenya; Medical Research Council Unit, The Gambia at the London School of Hygiene & Tropical Medicine, Banjul, The Gambia; Centre pour le Developpement des Vaccins (CVD-Mali), Bamako, Mali; Medical Research Council Unit, The Gambia at the London School of Hygiene & Tropical Medicine, Banjul, The Gambia; Medical Research Council Unit, The Gambia at the London School of Hygiene & Tropical Medicine, Banjul, The Gambia; Medical Research Council Unit, The Gambia at the London School of Hygiene & Tropical Medicine, Banjul, The Gambia; Division of Global Health Protection, US Centers for Disease Control and Prevention, Nairobi, Kenya; Division of Global Health Protection, US Centers for Disease Control and Prevention, Nairobi, Kenya; Division of Infectious Diseases and International Health, Department of Medicine, University of Virginia, Charlottesville, Virginia, USA; Division of Infectious Diseases and International Health, Department of Medicine, University of Virginia, Charlottesville, Virginia, USA; School of Public Health, Qingdao University, Qingdao, China; Division of Infectious Diseases and International Health, Department of Medicine, University of Virginia, Charlottesville, Virginia, USA; Center for Vaccine Development and Global Health, University of Maryland School of Medicine, Baltimore, Maryland, USA; Department of Medicine, University of Maryland School of Medicine, Baltimore, Maryland, USA; Department of Pediatrics, University of Maryland School of Medicine, Baltimore, Maryland, USA; Center for Vaccine Development and Global Health, University of Maryland School of Medicine, Baltimore, Maryland, USA; Department of Medicine, University of Maryland School of Medicine, Baltimore, Maryland, USA; Department of Pediatrics, University of Maryland School of Medicine, Baltimore, Maryland, USA; Center for Vaccine Development and Global Health, University of Maryland School of Medicine, Baltimore, Maryland, USA; Department of Medicine, University of Maryland School of Medicine, Baltimore, Maryland, USA

**Keywords:** *Salmonella*, diarrhea, Africa, children, antibiotic resistance

## Abstract

**Background:**

Non-typhoidal *Salmonella* (NTS) is a common cause of gastroenteritis in young children, with limited data on NTS serovars and antimicrobial resistance in Africa.

**Methods:**

We determined the prevalence of *Salmonella* spp. and frequency of antimicrobial resistance among serovars identified in stools of 0–59 month-old children with moderate-to-severe diarrhea (MSD) and controls enrolled in the Vaccine Impact on Diarrhea in Africa (VIDA) Study in The Gambia, Mali, and Kenya in 2015–2018, and compared with data from the Global Enteric Multicenter Study (GEMS; 2007–2010) and the GEMS-1A study (2011). *Salmonella* spp. was detected by quantitative real-time PCR (qPCR) and culture-based methods. Identification of serovars was determined by microbiological methods.

**Results:**

By qPCR, the prevalence of *Salmonella* spp. among MSD cases was 4.0%, 1.6%, and 1.9% and among controls was 4.6%, 2.4%, and 1.6% in The Gambia, Mali, and Kenya, respectively, during VIDA. We observed year-to-year variation in serovar distribution and variation between sites. In Kenya, *Salmonella enterica* serovar Typhimurium decreased (78.1% to 23.1%; *P* < .001) among cases and controls from 2007 to 2018, whereas serogroup O:8 increased (8.7% to 38.5%; *P* = .04). In The Gambia, serogroup O:7 decreased from 2007 to 2018 (36.3% to 0%; *P* = .001) but *S. enterica* serovar Enteritidis increased during VIDA (2015 to 2018; 5.9% to 50%; *P* = .002). Only 4 *Salmonella* spp. were isolated in Mali during all 3 studies. Multidrug resistance was 33.9% in Kenya and 0.8% in The Gambia across all 3 studies. Ceftriaxone resistance was only observed in Kenya (2.3%); NTS isolates were susceptible to ciprofloxacin at all sites.

**Conclusions:**

Understanding variability in serovar distribution will be important for the future deployment of vaccines against salmonellosis in Africa.

In high-income countries, non-typhoidal *Salmonella* (NTS) primarily causes a mild and self-limiting acute enterocolitis in otherwise healthy children [1, 2]. In contrast, in sub-Saharan Africa, NTS has emerged as a cause of severe invasive disease (iNTS) that disproportionately affects infants and young children who are immunocompromised, malnourished, or have had recent malaria [3]. The importance of NTS as a cause of diarrheal disease in sub-Saharan Africa is not well described and reports on the burden are conflicting. According to the 2015 Global Burden of Disease Study, 8.4% of the 446 000 diarrhea-related fatalities worldwide were due to NTS among children younger than 5 years [1].

The Global Enteric Multicenter Study (GEMS) across 7 sites in sub-Saharan Africa and South Asia used culture methods to isolate *Salmonella* spp. from stool and determined that NTS was significantly associated with moderate-to-severe diarrhea (MSD) in children aged younger than 5 years, but only at the Bangladesh and Kenya sites [4]. In GEMS, the prevalence of NTS in stools in Africa was 5.3% or less [5], similar to other reports from the African continent [6–9]. The ST313 sequence type of *Salmonella enterica* serovar Typhimurium (serovar Typhimurium) [10], a causal agent of iNTS in sub-Saharan Africa, was the only genotype recovered in the stools of Kenyan children [5]. A re-analysis of GEMS stools found that quantitative molecular testing increased the attributable fractions of MSD due to *Salmonella* spp. at all GEMS sites [11].

The Vaccine Impact on Diarrhea in Africa (VIDA) Study determined the incidence, etiology, and clinical consequences of diarrheal disease following introduction of rotavirus vaccine among children 0–59 months of age living in The Gambia, Mali, and Kenya using the same clinical, epidemiological, and microbiological methods as GEMS, except that quantitative real-time polymerase chain reaction (qPCR) was also used contemporaneously to detect pathogens. In VIDA, *Salmonella* spp. were significantly associated with MSD in 24- to 59-month-old children in The Gambia and Kenya by qPCR (unpublished data). Additionally, the adjusted attributable fraction of *Salmonella* spp. associated with MSD decreased from GEMS to VIDA.

Data on antibiotic resistance and seasonal patterns of NTS in sub-Saharan Africa are limited. While antimicrobial resistance (AMR) patterns have been investigated in some parts of sub-Saharan Africa, no systematic studies using identical methodology at multiple sites have been performed over an extended time frame [12–16]. Moreover, few studies have assessed resistance to commonly utilized antibiotics over time. Additionally, there is a paucity of information regarding the potential seasonal trends of diarrhea-associated NTS in sub-Saharan Africa.

To address these knowledge gaps regarding NTS diarrhea, we examined the features of *Salmonella* spp. identified in GEMS (2007–2010), a follow-up study called GEMS-1A (2011), and VIDA (2015–2018). The aim here was to describe the seasonality of *Salmonella* spp. and positivity by age group at each site as well as to determine the prevalence and distribution of *S. enterica* serovars and antibiotic susceptibility patterns among isolates from 2007 to 2018. We also examined the sensitivity and specificity of detection by culture versus qPCR at each of the sites to understand site-to-site variability in stool culture.

## METHODS

### Recruitment of Study Participants

VIDA, a prospective population-based case-control study among children belonging to censused populations at each site, was conducted 1–2 years following rotavirus vaccine introduction at the study sites, while GEMS and GEMS-1A were performed before rotavirus vaccine introduction. VIDA was implemented at 3 of the sites that participated in GEMS and GEMS-1A (Bamako, Mali; Basse, The Gambia; and Siaya County, Kenya), and so the analyses herein are limited to those sites. In The Gambia, an adjacent census site, Bansang, was added to Basse for VIDA to meet sample-size requirements. Enrollment occurred for 36 months at each site, from 11 May 2015 through 14 May 2018 in The Gambia and from 11 May 2015 through 18 May 2018 in Mali and from 27 July 2015 through 26 July 2018 in Kenya. The clinical, epidemiological, microbiological, and statistical methods are described in detail elsewhere for GEMS [17–19], GEMS-1A [20], and VIDA (unpublished data) [21]. In short, GEMS, GEMS-1A and VIDA enrolled children aged 0–59 months with MSD who attended a sentinel health center (SHC). MSD was defined as an episode of diarrhea (passage of ≥3 abnormally loose/watery stools per day) that was new (after ≥7 diarrhea-free days) and acute (duration <7 days) in a child with at least 1 of the following: bloody diarrhea, dehydration (poor skin turgor, sunken eyes, or required intravenous fluids), or recommended hospital admission. Enrollment was capped at approximately 9 children every 2 weeks per age stratum per site. Within 14 days of enrollment of an MSD case, 1 to 3 control children were randomly selected from the census database matched to the case by community, age, and sex, according to predefined criteria and considered eligible for enrollment if they had been diarrhea-free the previous 7 days (unpublished data) [21].

### Stool Collection and Transport

Stool specimens (collected from cases and controls) or rectal swabs (collected from cases with MSD who were to receive antibiotic treatment at the SHC) were inoculated into buffered glycerol saline (BGS) and Cary-Blair stool transport media and placed in refrigerated coolers. Stools were processed at local laboratories within 18 hours of collection.

### Identification of *Salmonella* spp. by Conventional Culture and Serotyping Methods

Identification of enteric agents in stools from all cases and controls followed GEMS methodologies, as previously described [18]. All *Salmonella* spp. isolated from stool cultures at the sites were sent to the Center for Vaccine Development and Global Health (CVD) at the University of Maryland School of Medicine for further characterization (for GEMS, GEMS-1A, and VIDA). At the CVD, *Salmonella* serovars and serogroups were identified by agglutination with antisera or by PCR, as previously described [5, 22, 23]. Serogrouping was performed on all isolates and full serotyping (for O and H antigens) was performed on O:4 isolates to identify serovar Typhimurium and O:9 isolates to identify serovars Enteritidis and Typhi. Any O:4 isolates that were not identified as serovar Typhimurium were designated as O:4. No serovar I 4,[5],12:i:- isolates were identified during VIDA. Any O:9 isolates that were not identified as serovars Typhi or Enteritidis were designated as O:9. Multilocus sequence types (MLSTs) of serovar Typhimurium isolates were determined by PCR and sequencing [24].

### Identification of Pathogens in Stool by qPCR

All VIDA stools collected from cases of MSD and 1 matched control for each case underwent qPCR-based TaqMan Array Card (TAC) testing to identify *Salmonella* spp. along with a standard array of pathogens, as described previously [11]. Notably, qPCR detected *Salmonella* spp. only at the species level and could not distinguish NTS from *S. enterica* serovar Typhi (serovar Typhi) or determine serovars; qPCR results were designated as *Salmonella* spp.

### Seasonality

We examined the seasonality of NTS during VIDA using qPCR data. In The Gambia and Kenya, both rainfall and temperature data were routinely collected and were available for the study time period. The meteorological department of Kenya collected daily total rainfall and maximum and minimum ambient temperature via stations located at the Kisumu International Airport. Similar government-operated meteorological stations in Basse and Bansang in The Gambia collected monthly summaries of both rainfall and ambient temperature. We were not able to obtain such data for Bamako, Mali, and instead utilized an online resource that provided monthly averages using data collected from 1982 to 2012 [25].

### Antimicrobial Susceptibility Testing

The susceptibility of *Salmonella* spp. isolated from stools during GEMS-1A and VIDA was evaluated for 5 commonly available antibiotics (ampicillin, ceftriaxone, chloramphenicol, trimethoprim-sulfamethoxazole [SXT] and ciprofloxacin) using the Kirby-Bauer disk diffusion method. Susceptibility of GEMS NTS isolates has been determined previously using the same methodology [5]. Multidrug resistance (MDR) was defined as resistance to chloramphenicol, SXT, and ampicillin [26]. Bacterial disk diffusion results were interpreted according to Clinical and Laboratory Standards Institute (CLSI) 2018 guidelines [27]. We examined AMR stratified by serovar to determine which serovars contributed to the prevalence of isolates resistant to individual antibiotics or multiple antibiotics at certain sites.

### Data Analysis

To compare the detection of *Salmonella* spp. obtained by stool culture versus qPCR, only specimens for which data from both the stool culture and qPCR assay were available were included; data were stratified by cases versus controls. To examine seasonality, since study enrollment is capped, we estimated the number of *Salmonella* spp. qPCR-positive cases that could have been seen during each month of VIDA using a site, age group, and calendar month weight composed of the total number of children with MSD presenting at an SHC divided by the total number of MSD cases enrolled. Significant changes in serovar prevalence over time were determined using the Cochran Armitage test.

### Ethical Review

This project was approved by the institutional review boards (IRBs) of the University of Maryland, Baltimore, Maryland, USA (HP-00062472); the US Centers for Disease Control and Prevention (CDC), Atlanta, Georgia, USA (reliance agreement, CDC protocol #6729); The Gambia Government/Medical Research Council/Gambia at the London School of Hygiene & Tropical Medicine (1409); the Comité d'Ethique de la Faculté de Médecine, de Pharmacie, et d'Odonto-Stomatologie, Bamako, Mali (no number); and the Kenya Medical Research Institute Scientific & Ethics Review Unit in Siaya County, Kenya (SSE 2996). Written, informed consent was obtained from the parent or primary caretaker of each child who met eligibility criteria before any research activities were performed.

## RESULTS

### VIDA Enrollment

**Figure 1.**
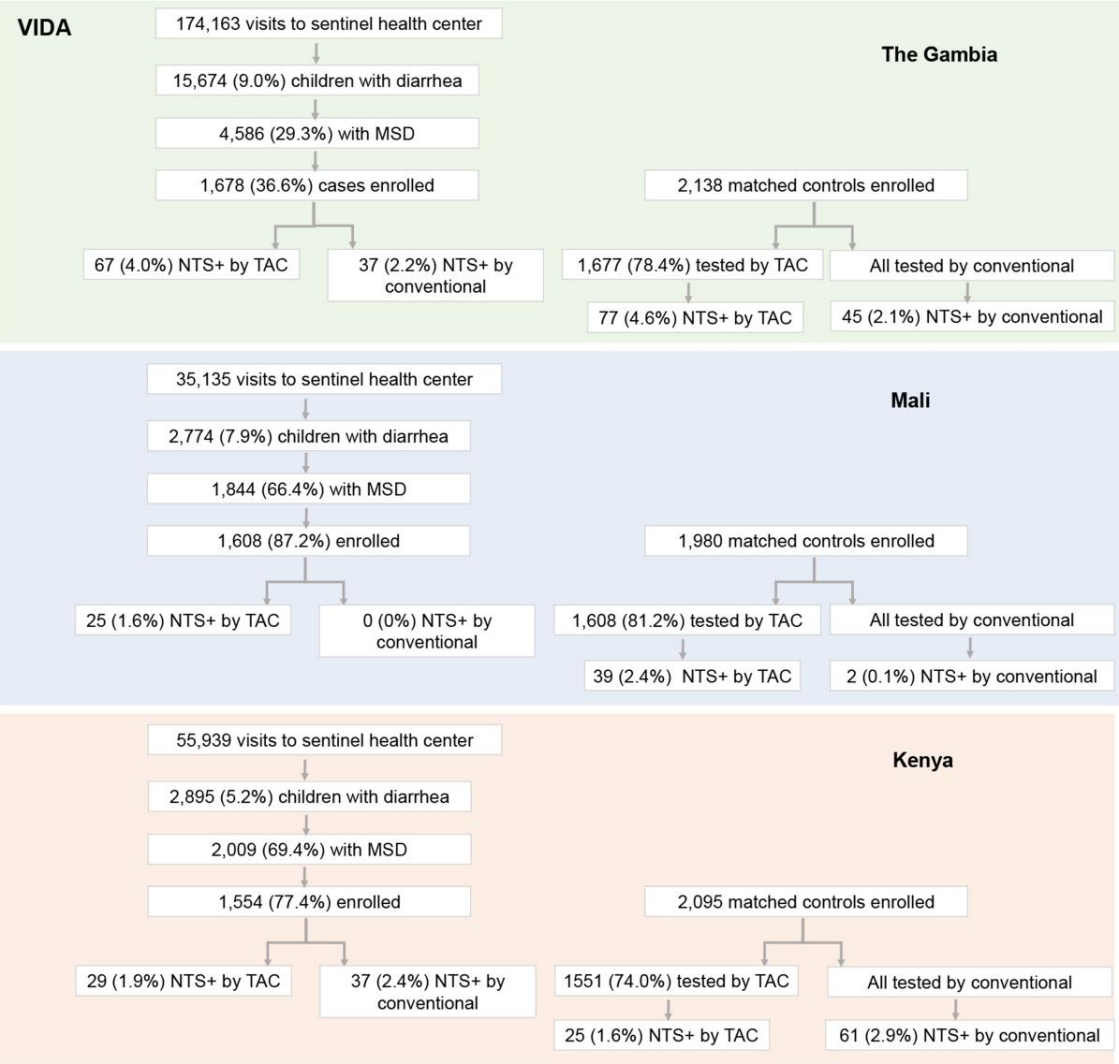
Schematic showing enrollment of study participants during VIDA. Children aged 0–59 months living in the study site census area who presented to sentinel health centers experiencing MSD were eligible to be enrolled in the study. For each case enrolled, 1–3 control children matched for age, sex, and residence were randomly selected from the census population and enrolled within 14 days of the index case. Abbreviations: MSD, moderate-to-severe diarrhea; NTS, non-typhoidal *Salmonella*; TAC, TaqMan Array Card; VIDA, Vaccine Impact on Diarrhea in Africa.

A total of 11 053 children aged 0–59 months were enrolled in the VIDA study, including 4840 MSD cases and 6123 controls (Figure 1).

### Prevalence and Seasonal Trends of *Salmonella* spp.

By qPCR, prevalence rates among cases were 4.0%, 1.6%, and 1.9% in The Gambia, Mali, and Kenya, respectively, and 4.6%, 2.4%, and 1.6% among controls, respectively (Figure 1). By conventional microbiology, prevalence rates of *Salmonella* spp. in the stool of cases were 2.2%, 0%, and 2.4% in The Gambia, Mali, and Kenya, respectively, and 2.1%, 0.1%, and 2.9% among controls, respectively. The distribution of *Salmonella* spp. was similar across age groups, with 2.4%, 2.1%, and 3.0% detected by qPCR and 1.5%, 1.4%, and 1.7% detected by culture from 0–11-, 12–23-, and 24–59-month-old children, respectively, when all sites were combined (Table 1). In 24–59-month-old children in The Gambia and Kenya where *Salmonella* spp. was associated with MSD, qPCR detected *Salmonella* spp. in 5.6% of cases and 3.7% of controls in The Gambia and 1.1% of cases and 0.9% of controls in Kenya. Compared with qPCR, culture showed excellent specificity (99.7%) but poor sensitivity (22.9%) (Supplementary Table 1). Among cases, we observed sensitivities of culture to be 37.3% for The Gambia, 0% for Mali, and 62.1% for Kenya.

**Table 1.** *Salmonella* spp. Positivity in Cases and Controls Using Stool Culture and qPCR by Site and Age Group in VIDA

	*Salmonella* spp. Positivity by Site, n/N (%)							
	All Sites		The Gambia		Mali		Kenya	
Assay and Age Group	Cases	Controls	Cases	Controls	Cases	Controls	Cases	Controls
qPCR								
ȃ0–11 months	42/1720 (2.4%)	66/1719 (3.8%)	18/540 (3.3%)	33/539 (6.1%)	8/595 (1.3%)	16/595 (2.7%)	16/585 (2.7%)	17/585 (2.9%)
ȃ12–23 months	36/1695 (2.1%)	40/1696 (2.4%)	20/617 (3.2%)	25/618 (4.0%)	8/552 (1.4%)	11/552 (2.0%)	8/526 (1.5%)	4/526 (0.8%)
ȃ24–59 months	43/1421 (3.0%)	35/1421 (2.5%)	29/520 (5.6%)	19/520 (3.7%)	9/461 (2.0%)	12/461 (2.6%)	5/440 (1.1%)	4/440 (0.9%)
Culture								
ȃ0–11 months	26/1721 (1.5%)	58/2111 (2.7%)	10/540 (1.9%)	19/696 (2.7%)	0	1/690 (0.1%)	16/586 (2.7%)	38/725 (5.2%)
ȃ12–23 months	24/1698 (1.4%)	21/2130 (1.0%)	12/618 (1.9%)	14/748 (1.9%)	0	0	12/528 (2.3%)	7/726 (1.0%)
ȃ24–59 months	24/1421 (1.7%)	29/1972 (1.5%)	15/520 (2.9%)	12/694 (1.7%)	0	1/634 (0.2%)	9/440 (2.0%)	16/644 (2.5%)

Abbreviations: qPCR, quantitative real-time polymerase chain reaction; VIDA, Vaccine Impact on Diarrhea in Africa.

**Figure 2.**
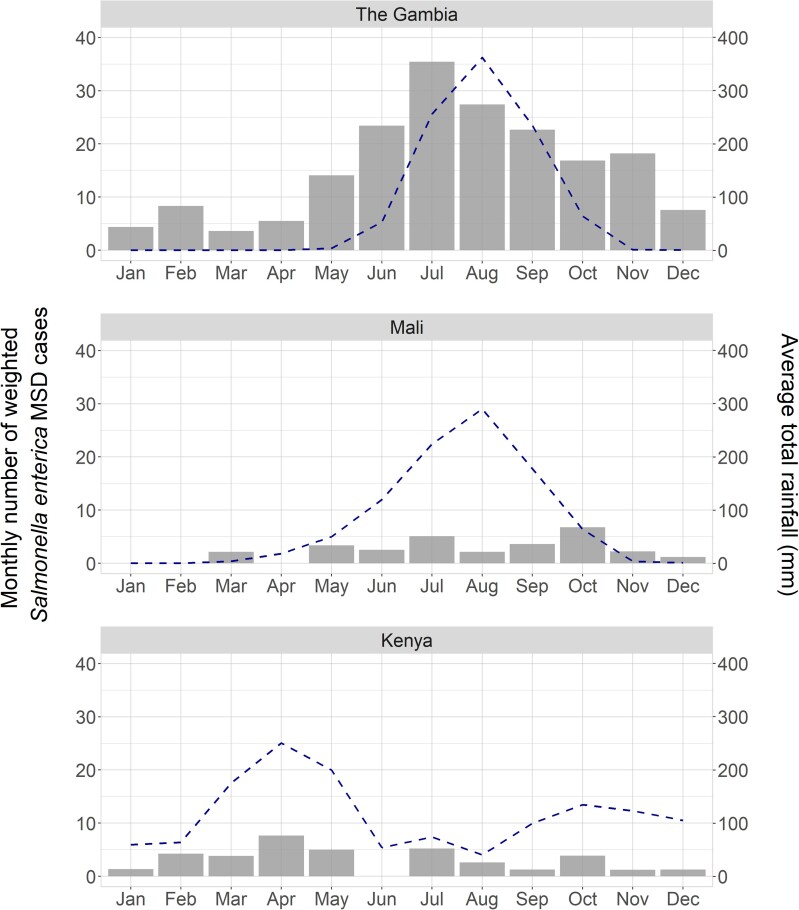
Seasonal distribution of *Salmonella* spp. at study sites during VIDA. Shown are the weighted proportions of monthly aggregate *Salmonella* spp.–positive stools detected by qPCR. The weighted number of *Salmonella* spp.–positive cases, shown in bars, represents the total number with MSD seeking care at the sentinel health centers at each site where the study was conducted, divided by the total number enrolled using the study-specific sampling strategy, multiplied by the number of positive *Salmonella* cases (by age group, site, and month). Monthly rainfall in millimeters is depicted by a dashed line. Abbreviations: MSD, moderate-to-severe diarrhea; qPCR, quantitative real-time polymerase chain reaction; VIDA, Vaccine Impact on Diarrhea in Africa.

The qPCR data from The Gambia suggest that peak *Salmonella* spp. detection coincides with peak rainfall; however, due to the low number of *Salmonella* spp.–positive cases at the Mali and Kenya sites, we could not conclusively determine seasonal effects (Figure 2).

### Prevalence of *Salmonella* Serovars Identified by Culture in VIDA

Serotyping showed a variety of serovars and serogroups isolated from cases and controls (Table 2). Serovar Typhimurium (8/1554 [0.5%]) and serogroup O:4 serovars other than Typhimurium (9/1554 [0.6%]) at the Kenya site and serogroup O:13 (G; 13/1678 [0.8%]) at The Gambia site were the most prevalent serogroups/serovars among cases. Of the 11 serovar Typhimurium isolates cultured from VIDA stools, 9 were ST313 and 2 were ST36. Only 2 serovar Typhi were identified.

**Table 2.** Prevalence of *Salmonella enterica* Serovars Cultured From Stools During VIDA in The Gambia and Kenya

	Prevalence of Serovars by Site			
	The Gambia		Kenya	
*Salmonella* Serovar or Serogroup	Cases	Controls	Cases	Controls
No. of participants	1678	2138	1554	2095
Typhimurium	0	0	8 (0.5%)	3 (0.1%)
Enteritidis	0	10 (0.5%)	5 (0.3%)	2 (0.1%)
Typhi	0	0	1 (0.1%)	1 (0%)
O:13	13 (0.8%)	5 (0.2%)	1 (0.1%)	1 (0%)
O:9; O:3,10; O:11	7 (0.4%)	8 (0.4%)	3 (0.2%)	20 (1.0%)
O:8	2 (0.1%)	2 (0.1%)	5 (0.3%)	12 (0.6%)
O:7	2 (0.1%)	5 (0.2%)	5 (0.3%)	7 (0.3%)
O:4	7 (0.4%)	6 (0.3%)	9 (0.6%)	8 (0.4%)
Other serovars	6 (0.4%)	9 (0.4%)	1 (0.1%)	7 (0.3%)
Total	37 (2.2%)	45 (2.1%)	38 (2.4%)^a^	61 (2.9%)

Data are presented as n (%) unless otherwise indicated. Abbreviation: VIDA, Vaccine Impact on Diarrhea in Africa.

Thirty-eight *S. enterica* strains isolated from 37 children; 1 child had 2 serovars isolated.

### Distribution of *Salmonella* Serovars Identified Using Stool Culture Methods From 2008 to 2018

**Figure 3.**
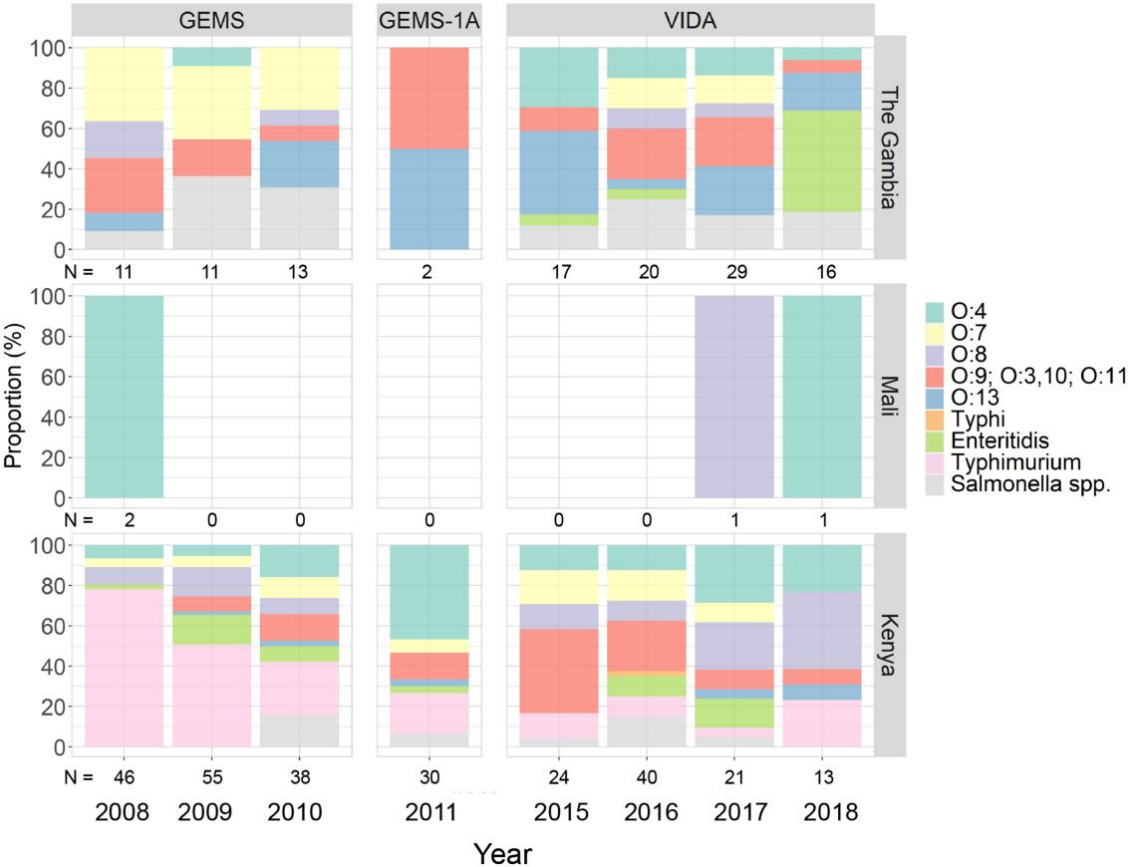
Annual proportions of *Salmonella enterica* serovars identified in stools from cases and controls during GEMS, GEMS-1A, and VIDA. *Salmonella* spp. identified by conventional culture methods were serotyped to identify serogroup or serotypes. Abbreviations: GEMS, Global Enteric Multicenter Study; VIDA, Vaccine Impact on Diarrhea in Africa.

Collectively, only 4 isolates were recovered in the stools of participants at the Mali site during all 3 studies. Overall proportions of serovar Typhimurium decreased from the start of GEMS through VIDA in Kenya (78.3% in 2008 to 23.1% in 2018; *P* < .001) (Figure 3). Conversely, there was an increase in serogroup O:8 in Kenya during this time (8.7% in 2008 to 38.5% in 2018; *P* = .04). In The Gambia, serogroup O:7 significantly decreased from the start of GEMS through VIDA (36.3% in 2008 to 0% in 2018; *P* = .001) and there was an increase in serovar Enteritidis during VIDA (5.9% in 2015 to 50% in 2018; *P* = .002). Serovar Typhimurium was only detected in stools of Kenya participants and not at the other sites throughout all studies.

### Evaluating Antimicrobial Susceptibility of NTS Isolates Across 3 Studies

**Figure 4.**
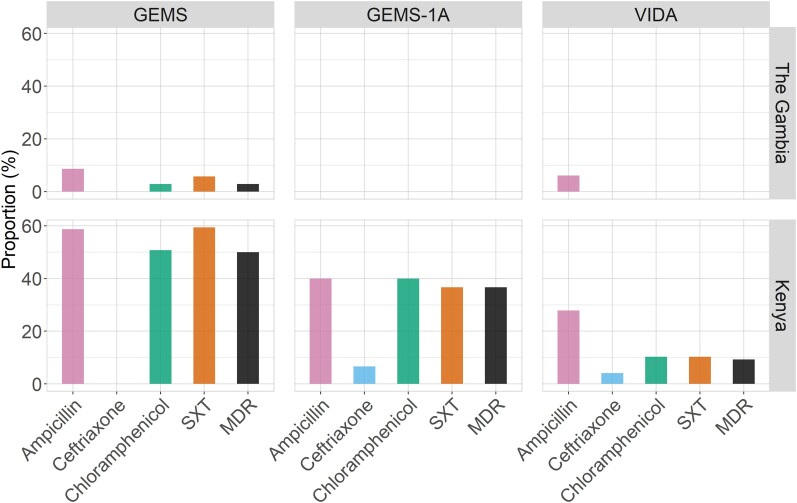
Percentage of *Salmonella enterica* serovars from cases and controls that demonstrated antimicrobial resistance during GEMS, GEMS-1A, and VIDA. *Salmonella* spp. recovered during GEMS, GEMS-1A, and VIDA studies were tested for susceptibility to 5 common antimicrobial drugs and shown is the proportion of resistant isolates detected in stools at The Gambia and Kenya. Note that resistance to ciprofloxacin was not detected. Abbreviations: GEMS, Global Enteric Multicenter Study; MDR, multidrug-resistant; SXT, trimethoprim-sulfamethoxazole; VIDA, Vaccine Impact on Diarrhea in Africa.

We evaluated 176, 32, and 182 NTS isolates (from cases and controls) recovered during GEMS, GEMS-1A, and VIDA, respectively, for susceptibility to antibiotics (serovar Typhi was excluded). Of 119 NTS isolates recovered from The Gambia during all 3 studies, less than 10% were resistant to ampicillin, chloramphenicol, or SXT; the proportion of isolates that showed MDR was 1 of 119 (0.8%) (Figure 4). Multidrug resistance among NTS isolates for which AMR data were available at the Kenya site was 69 of 138 (50.0%) in GEMS, 11 of 30 (36.7%) in GEMS-1A, and 9 of 97 (9.3%) in VIDA. We did not detect ciprofloxacin resistance at any of the 3 African sites during GEMS, GEMS-1A, or VIDA (Figure 4; ciprofloxacin not shown); however, resistance to ceftriaxone was detected from 2011 in Kenya (6/265 [2.3%] across all 3 studies).

**Figure 5.**
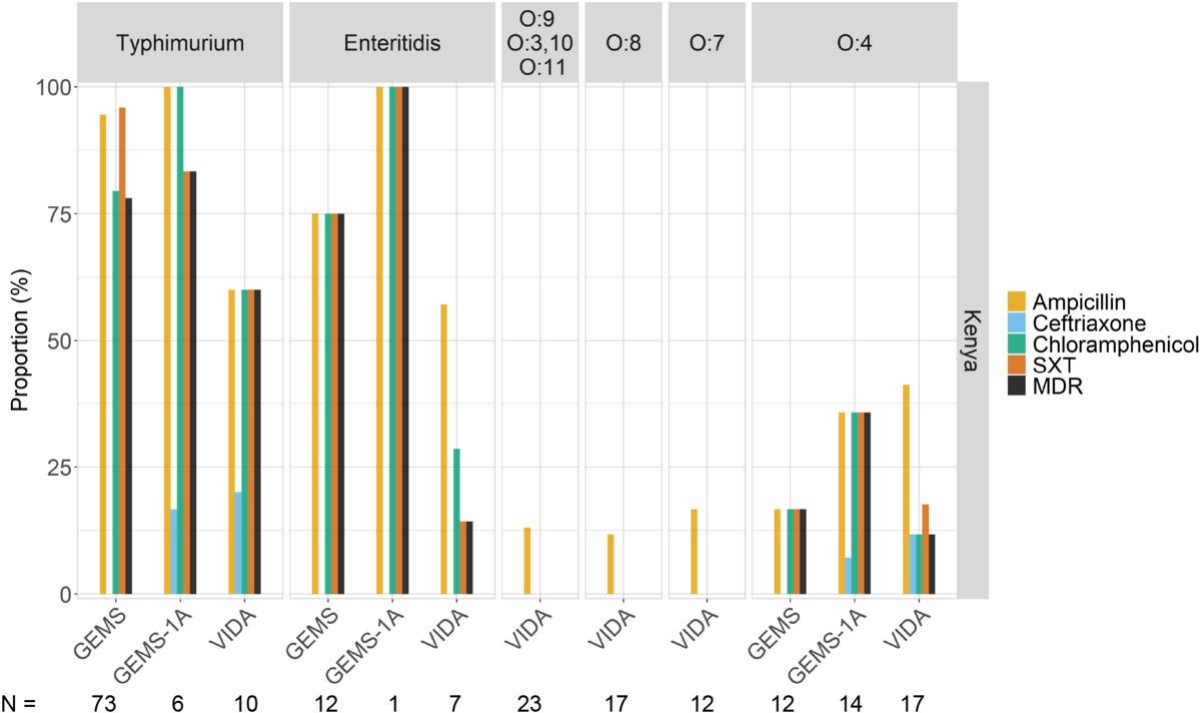
Percentage of *Salmonella* spp. from cases and controls during GEMS, GEMS-1A, and VIDA at the Kenya site with antimicrobial resistance, by serovar. Only 3 serovars or serogroups were found to be resistant to any of the drugs tested during all the study periods. Note that resistance to ciprofloxacin was not detected. Abbreviations: GEMS, Global Enteric Multicenter Study; MDR, multidrug-resistant; SXT, trimethoprim-sulfamethoxazole; VIDA, Vaccine Impact on Diarrhea in Africa.

The frequencies of MDR (among all 3 studies) stratified by serovar in Kenya were as follows: 68 of 89 (76.4%) for serovar Typhimurium; 11 of 20 (55%) for serovar Enteritidis; 9 of 43 (20.9%) for serogroup O:4 isolates (other than serovar Typhimurium); 0 of 36 (0%) for serogroups O:9, O:3,10, and O:11 combined; 0 of 32 (0%) for O:8; and 0 of 23 (0%) for O:7 (Figure 5). All 82 antibiotic-resistant serovar Typhimurium strains were ST313; 2 ST36 isolates detected during VIDA were susceptible to all antibiotics in our panel. The majority of serovar Typhimurium isolates (all ST313) collected in stools at the Kenya site during GEMS (57/73 [95.9%]), GEMS-1A (5/6 [83.3%]), and VIDA (6/10 [60.0%]) were MDR (Figure 5).

## DISCUSSION

The key findings of our study are that *Salmonella* spp. is not common in the stool of young children in sub-Saharan Africa and that there is site-to-site and year-to-year variability in serovar distribution. We observed low to moderate proportions of antibiotic-resistant NTS but certain serovars showed high proportions of MDR.

We observed a low prevalence of *Salmonella* spp. among cases and controls as we and others have previously reported [5,8,9,28–30]. Controls living in the same vicinity as cases may have been exposed to *Salmonella* spp. but did not experience diarrhea, or had recovered from recent salmonellosis [29]. As a result, *Salmonella* spp. was not significantly associated with MSD in any age stratum or site, except for 24–59-month-olds in The Gambia and Kenya during VIDA (unpublished data). The prevalence of each serovar was similar among cases and controls in The Gambia and Kenya, suggesting that there was no particular serovar associated with disease.

When we assessed the effect of weather on salmonellosis, we observed that *Salmonella* spp.–positive cases were more frequent during the rainy season in The Gambia but had no seasonal pattern in Kenya or Mali. Other studies reported that iNTS in the Democratic Republic of Congo [31], Malawi [32], and Burkina Faso [33] was most frequently isolated in the rainy seasons concomitantly with malaria. The lack of clear understanding as to whether seasonality influenced the frequency of fecal *Salmonella* spp. detection in Mali and Kenya is most likely due to the low qPCR prevalence that we observed at these study sites during VIDA. Combined with the data showing the presence of NTS in controls, and the fact that NTS from stool and blood is highly related genetically [5, 34–36], the data suggest that there may be more NTS circulating in the community during the rainy season, which results in increased iNTS cases.

Upon assessing the distribution of *Salmonella* serovars isolated during 3 different time periods, we found some shifts in the abundance of certain serovars. In Kenya, serovar Typhimurium was the most abundant serovar recovered in stools during GEMS, but it decreased in proportion to other serovars throughout VIDA. Similarly, serovar Typhimurium is the most abundant serovar isolated in bloodstream infections in this part of Kenya but has also been declining over time [37]. Serogroup O:7 was prevalent in The Gambia during GEMS but not VIDA. Interestingly, serovar Enteritidis was the dominant serovar isolated in The Gambia in 2018, although it was rarely isolated in our studies prior to 2018. A previous study conducted in this same site in The Gambia found that serovar Enteritidis was the dominant serovar isolated from blood and/or cerebrospinal fluid from children aged 2–29 months with iNTS [38]. The unstable or cyclical distribution of serovars Enteritidis and Typhimurium has been reported in regions of sub-Saharan Africa experiencing iNTS [39–42]. Taken together, our data show that the distribution of *Salmonella* serovars in stool varies considerably from year to year and between geographic sites, which has implications for future intervention studies, such as vaccines that target particular serovars. The factors driving these shifts in serovar distribution are unknown.

We observed a reduction from 2007 to 2018 in the proportion of *Salmonella* spp. that were resistant to antibiotics in Kenya; however, AMR at this site was driven by specific serovars. As shown in Figure 5, serovars Typhimurium and Enteritidis were frequently resistant to antibiotics, whereas other serovars, such as those belonging to serogroup O:4, were more susceptible. The high prevalence of AMR observed in GEMS was therefore driven by the high proportion of serovar Typhimurium during 2008–2010. Although some resistance to ampicillin was observed at The Gambia site, it was much lower than in Kenya. Additionally, unlike isolates from the Kenya site, isolates from Gambian children's stools were highly susceptible to chloramphenicol and SXT. The low resistance of NTS to ampicillin, SXT, and chloramphenicol in The Gambia has also been reported among NTS isolates collected from Guinea Bissau and Senegal [8] and from the Central African Republic [6]. Collectively, these data suggest a regional distribution of AMR that is driven by serovar distribution and that resistant NTS could be prevented by targeting certain serovars (eg, through vaccines).

Although antibiotic therapy is generally not recommended for simple NTS gastroenteritis in otherwise healthy children, antibiotic treatment would be recommended for iNTS, which can sometimes include diarrhea [3]. In 2011, we detected nascent but low resistance to ceftriaxone at the Kenya site, potentially suggesting an increase in the availability and/or use of ceftriaxone at the site. Ceftriaxone is often used when parenteral therapy is required for *Salmonella* spp. infections. Ceftriaxone-resistant isolates have also been cultured from iNTS in the same region of Kenya, suggesting that these bacteria are circulating there and may become a significant public health threat in the future [43]. Resistance to ceftriaxone has emerged among NTS and is increasing in parts of South Asia, such as Pakistan, and controlling these infections will only become more difficult [44]. Of note, all isolates recovered in stools during GEMS, GEMS-1A, and VIDA at study sites in sub-Saharan Africa were susceptible to ciprofloxacin, which is a first-line therapy for the treatment of *Shigella*. The complete lack of resistance to ciprofloxacin was also noted among *Shigella* spp. isolated and characterized during the 3 studies [45].

The main limitation of our study was the low prevalence of *Salmonella* spp. at the Mali site. Mali also had the lowest sensitivity in terms of detection by culture compared with The Gambia and Kenya. Given that qPCR detected *Salmonella* spp.–positive stools at higher frequencies, the low bacterial recovery was possibly due to the common use of antibiotics for children with diarrhea at our sites [46] and possibly suboptimal stool transport or culture techniques at the sites despite intensive quality-control activities. We think that the superior detection by qPCR was due to increased sensitivity and not false positives. Similar observations have been noted for *Shigella* spp. in VIDA [45]; *Shigella* spp. are also difficult to culture from stool. Other limitations include the following: gaps in the data (2012–2014); incomplete years, which can affect the prevalence by year; limited data to assess seasonality; expanding the study area in The Gambia between GEMS and VIDA; and that qPCR did not provide serovar identity.

There are 4 main conclusions from our study: (1) the distribution of *Salmonella* serovars varied considerably over time and by geographic location; (2) certain serovars show high proportions of MDR; (3) NTS from Kenya has acquired resistance to ceftriaxone; however, NTS isolates from VIDA sites are susceptible to ciprofloxacin; and (4) peak detection of *Salmonella* spp. coincided with the rainy season in The Gambia. Continued surveillance in this region will be needed to monitor AMR among *Salmonella* spp. to understand the spread of AMR, to guide antibiotic prescription and use in sub-Saharan Africa, and to inform the development and deployment of vaccines against NTS.

## Supplementary Data

Supplementary materials are available at *Clinical Infectious Diseases* online. Consisting of data provided by the authors to benefit the reader, the posted materials are not copyedited and are the sole responsibility of the authors, so questions or comments should be addressed to the corresponding author.

## Supplementary Material

ciac985_Supplementary_Data

## Notes

***Acknowledgments*.** The authors express their deep gratitude to the families who participated in these studies, the clinical and field staff for their exceptional hard work and dedication, and to the physicians, administration, and health officials at every site who generously provided facilities and support for the conduct of the study. They are grateful to Catherine Johnson, Chris Focht, and Nora Watson at the Emmes Company, LLC, for expert data management and reporting. Special thanks go to Carl Kirkwood, Duncan Steele, and Anita Zaidi at the Bill & Melinda Gates Foundation for helpful oversight, and to the following members of the International Scientific Advisory Committee for providing insightful comments and guidance: Janet Wittes (Chair), George Armah, John Clemens, Christopher Duggan, Stephane Helleringer, Ali Mokdad, James Nataro, and Halvor Sommerfelt.

***Financial support*.** This work was supported by the Bill & Melinda Gates Foundation grants OPP1111236 and OPP1116751.

***Supplement sponsorship*.** This supplement was sponsored by the Bill & Melinda Gates Foundation. This study is based on research funded in part by grants from the Bill & Melinda Gates Foundation (OPP1111236/OPP1116751).
