# Supplementary material for: Prevalence of Salmonella in Stool During the Vaccine Impact on Diarrhea in Africa (VIDA) Study, 2015–2018
Source: Clin Infect Dis. 2023 Apr 19;76(Suppl 1):S87–96. doi: 10.1093/cid/ciac985 (PMC10116559; doi:10.1093/cid/ciac985)
Supplement: ciac985_Supplementary_Data [file ciac985_supplementary_data.pdf]

Supplementary Table 1. Sensitivity and specificity of stool culture versus qPCR by site in VIDA.

	**qPCR**											
	**The Gambia**				**Mali**				**Kenya**			
	**Cases**		**Controls**		**Cases**		**Controls**		**Cases**		**Controls**	
**Stool culture**	**Positive**	**Negative**	**Positive**	**Negative**	**Positive**	**Negative**	**Positive**	**Negative**	**Positive**	**Negative**	**Positive**	**Negative**
**Positive for *Salmonella***	25/37 (67.6%)	12/37 (32.4%)	10/27 (37.0%)	17/27 (63.0%)	0	0	0	1/1 (100.0%)	18/37 (48.6%)	19/37 (51.4%)	7/40 (17.5%)	33/40 (82.5%)
**Negative for *Salmonella***	42/ 1,624 (2.6%)	1582/ 1,624 (97.4%)	67/ 1637 (4.1%)	1570/ 1637 (95.9%)	25/ 1601 (1.6%)	1576/ 1601 (98.4%)	39/ 1599 (2.4%)	1560/ 1599 (97.6%)	11/ 1504 (0.7%)	1493/ 1504 (99.3%)	18/ 1496 (1.2%)	1478/ 1496 (98.8%)
**Sensitivity**	37.3%		13.0%		0		0		62.1%		28.0%	
**Specificity**	99.2%		98.9%		100%		99.6%		98.7%)		99.8%	
